# Lignin-degrading peroxidases in white-rot fungus *Trametes hirsuta* 072. Absolute expression quantification of full multigene family

**DOI:** 10.1371/journal.pone.0173813

**Published:** 2017-03-16

**Authors:** Daria V. Vasina, Konstantin V. Moiseenko, Tatiana V. Fedorova, Tatiana V. Tyazhelova

**Affiliations:** A.N. Bach Institute of Biochemistry, Research Center of Biotechnology of the Russian Academy of Sciences, 33, bld. 2 Leninsky Ave., Moscow, Russia; Helsingin Yliopisto, FINLAND

## Abstract

Ligninolytic heme peroxidases comprise an extensive family of enzymes, which production is characteristic for white-rot Basidiomycota. The majority of fungal heme peroxidases are encoded by multigene families that differentially express closely related proteins. Currently, there were very few attempts to characterize the complete multigene family of heme peroxidases in a single fungus. Here we are focusing on identification and characterization of peroxidase genes, which are transcribed and secreted by basidiomycete *Trametes hirsuta* 072, an efficient lignin degrader. The *T*. *hirsuta* genome contains 18 ligninolytic peroxidase genes encoding 9 putative lignin peroxidases (LiP), 7 putative short manganese peroxidases (MnP) and 2 putative versatile peroxidases (VP). Using ddPCR method we have quantified the absolute expression of the 18 peroxidase genes under different culture conditions and on different growth stages of basidiomycete. It was shown that only two genes (one MnP and one VP) were prevalently expressed as well as secreted into cultural broth under all conditions investigated. However their transcriptome and protein profiles differed in time depending on the effector used. The expression of other peroxidase genes revealed a significant variability, so one can propose the specific roles of these enzymes in fungal development and lifestyle.

## Introduction

Heme peroxidases are large group of biocatalysts with ecological and biotechnological significance. Ligninolytic class II secreted heme peroxidases (PODs) include manganese peroxidase (MnP; EC 1.11.1.13), lignin peroxidase (LiP; EC 1.11.1.14), and versatile peroxidase (VPs; EC 1.11.1.16) enzymes with broad organic and inorganic substrate range [1,2]. These enzymes are involved in bioconversion of various hardly degradable compounds and in transformation of humic substances and halogenated carbohydrates; however, participation in biodegradation of plant lignocelluloses and lignins is considered to be their main function [3,4]. Along with laccases and hydrogen peroxide-generating enzymes, PODs comprise typical lignin-degrading complex of basidiomycetes. While LiP is capable of unmediated oxidation of recalcitrant polymer, MnP act through mediated oxidation of phenolic intermediates [5]. In addition to direct participation in depolymerization of plant lignin, an ability to cleave most of the lignin indirectly, through low-molecular-weight oxidants that can enter the wood cell wall, is suggested for the peroxidases. LiPs are proposed to oxidize lignin with free radicals generated through oxidation of various secreted metabolites (e.g., veratryl alcohol) [6], and MnPs can produce reactive oxygen species by oxidation of fungal unsaturated fatty acids [7,8].

In the genomes of basidiomycetes, PODs are encoded by multigene families variable in size (from six genes in *Stereum hirsutum* to 26 genes in *Trametes versicolor*) [9]. Their presence within a genome is a distinctive feature of lignin-degrading white-rot fungi group, since polysaccharide-degrading brown-rot species lack these enzymes [9].

Promoter region of PODs contains a relatively conservative set of cis-elements [10] typical for ligninolytic enzymes, including HSE, MRE, CRE, XRE, SP1, and AP2 responsive elements. However, composition of these elements is markedly variable for each member of the multigene family even within one organism. It is known that media composition and fungal growth conditions differentially affect the production of PODs isozymes, their expression and secretion profiles. These facts indicate that the functions of PODs in fungi could be considerably more variable than participation in ligninolytic processes exclusively, and subfunctionalization of the members of multigene families is assumed [11].

A series of studies on evaluation of differential expression of PODs encoding genes in white-rot fungi currently exists. Researches were conducted on the influence of nitrogen and carbon sources, as well as various inducers, on peroxidases expression in *Phanerochaete chrisosporium*, *Pleurotus sp*., *Trametes trogii*, and *Phlebia radiata* [12–15] (for more information see [10]). On the basis of existing data, expression of only certain patterns of heme peroxidases rather than production of the whole set of isozymes is typical for basidiomycetes. For example, in *P*. *chrysosporium* nine out of sixteen transcripts of PODs encoding genes (six LiPs and three MnPs) were markedly elevated after the onset of extracellular oxidation in the cultures grown on spruce wood [13]. For basidiomycete *Pleurotus ostreatus* PC9, primary expression of two (out of nine) isozymes was typical, and only four peroxidases were secreted into cultural broth [12]. Currently, the last mentioned study remains the only attempt to characterize the whole PODs gene family. For peroxidases of *P*. *ostreatus* PC9, gene identity did not exceed 60% for nucleotide sequences, which allowed utilizing a common real-time PCR method based on SYBR-Green Dye for evaluation of their expression. However, utilization of real-time PCR for highly similar genes is rather restrictive [16]. The *T*. *versicolor* PODs genes (NCBI BioProject #PRJNA56097) possess a greater identity (in case of nucleotide sequences, genes with at least 80% identity can be found for the majority of the multigene family members), so one can propose that the conventional qPCR doesn’t fit such analyses in *Trametes* genus.

Droplet digital PCR (ddPCR) [17,18] is becoming a widely used method. This is a third-generation implementation of conventional PCR that has been attracting considerable attention due to multiple advantages, and has already been tested for numerous applications [19–21]. This approach allows the detection of small changes in fluorescence intensity by the instrument, comparing to a similar absolute amount of fluorescence detected using conventional qPCR platforms with TaqMan probes. In addition, absolute number of target nucleic acid molecules contained in the original sample before partitioning can be calculated directly from the ratio of positive events to total partitions, using binomial Poisson statistics [22,23]. Anticipating closely related gene sequences encoding peroxidase enzymes in *T*. *hirsuta*, accurate quantification of cDNA becomes crucial. The ddPCR method also allows to achieve reliable and reproducible measurements of small changes even for low abundance of mRNA, and to separate highly similar mRNAs.

In the present study, we characterized individual genes encoding class II peroxidases in *T*. *hirsuta* and analyzed the expression profiles of this multigene family. We used the ddPCR method to quantify the absolute expression of all annotated peroxidase genes on different growth stages of basidiomycete, and during introduction of effectors (alkali lignin and bromocresol green dye) into cultural broth. In addition, protein profiling of peroxidase complex secreted by fungi under these conditions was performed.

## Materials and methods

### Fungal strain and culture conditions

Mycelium of basidiomycete strain *Trametes hirsuta* 072 used in this study was received from the Collection of the Komarov Botanical Institute, Russian Academy of Sciences (St. Petersburg). Fungi were stored on wort-agar slants at 4°C. The static precultivation of mycelium was performed at 26–28°C in 750-ml Erlenmeyer flasks with 200 ml of glucose-peptone medium (GP) which contains glucose as a sole carbon source [24]. For further cultivation, the mycelium was grinded into small fragments using ceramic beads and inoculated into flasks. The submerged cultivation on GP media was performed at 26–28°C in Erlenmeyer flasks. The cultivation media was also supplied with the following agents: 2 g/l of alkali lignin (Sigma-Aldrich, USA) (further “AL” samples) and 100 mg/l of bromocresol green (Alfa Aesar, Germany) (further “BR” samples). To determine the dry weight of biomass, the fungus was cultivated at the specified conditions in individual 750-ml Erlenmeyer flasks for the defined time, harvested, and dried at 105°C. The weights were recorded when they achieved constant values after drying. The samples were taken with 12 h interval. Reducing sugars in culture broth were determined by the method of Bertrand based on the determination of aldehyde and ketone groups oxidized by Fehling’s reagent with the formation of Cu_2_O and its quantification [25]. The cultivations were performed in triplicate.

### *In silico* search of PODs genes in the genome of *T*. *hirsuta* and their characterization

Gene models in the genome of *T*. *hirsuta* 072 [26] were predicted using Augustus software [27]. All peroxidase domains containing proteins were subjected to BLASTp [28] search, and those that gave good peroxidase hits were selected.

The molecular weights (Mw) and isoelectric points (pI) of each putative peptide, as well as nucleic and amino acid (aa) sequences identity were predicted using the Sequence Manipulation Suite [29], ExPASy tools server [30], and BLAST algorithms [28]. The glycosylation sites were predicted by NetNGlyc 1.0 (http://www.cbs.dtu.dk/services/NetNGlyc/) and NetOGlyc 4.0 (http://www.cbs.dtu.dk/services/NetOGlyc/) [31].

### Phylogenetic tree construction

Multiple sequence alignment of nucleotide (CDS) and translated amino acid sequences were generated with the ClustalW program [32], and ambiguous alignment regions were removed using Gblocks (v 0.91b) [33]. Two phylogenetic trees were reconstructed based on the alignments of nucleotide and amino acid sequences.

For nucleotide sequences alignment, the best fitting model of sequence evolution was determined using jModelTest2 software [34] with 11 substitution schemes. Model selection was performed using the Akaike information criterion (AIC), and the GTR+G model was selected. The phylogenetic tree was constructed using the maximum likelihood (ML) method with the PhyML program (v 3.0) [35]. The reliability for the internal nodes was assessed using the bootstrapping method (100 bootstrap replicates).

For amino acid sequences alignment, phylogenetic tree was constructed using the maximum likelihood method implemented in the PhyML program (v 3.0) [35] under the LG model. Reliability for the internal nodes was assessed using the bootstrapping method (100 bootstrap replicates).

Both constructed trees were of the same topology. For the present study, graphical representation of phylogenetic tree based on the amino acids was obtained with ggtree (Yu G and Lam TTY [http://www.bioconductor.org/packages/ggtree]).

### RNA extraction and cDNA amplification

Samples for the RNA extraction and expression analysis were collected on the 3^rd^, 5^th^ and 8^th^ day of fungal cultivation on GP, AL and BR media. An extraction of total RNA with TRIzol Reagent (Life technologies, USA) from frozen mycelia samples was performed. The extracted nucleic acid solutions were treated with DNase I (Thermo Scientific, USA) for 30 min. The integrity and quantity of RNA were determined by spectrophotometer (Nanodrop ND-1000, LabTech International, ES, UK) and by 1.0% agarose gel electrophoresis. cDNA was obtained from the mRNA using MMLV RT KIT (Evrogen, Russia) with Oligo(dT) primers according to the manufacturer’s instructions. One μg of total RNA was used per reaction.

### Droplet digital PCR (ddPCR)

The peroxidase gene-specific primers and TaqMan probes are presented in S1 Table. Primers and probes design was conducted manually and primers were analyzed with Primer3 software [36]. For most genes TaqMan probes were constructed at the exon-intron junctions. The expression of *T*. *hirsuta* peroxidase genes was quantified using a QX100 droplet digital PCR system (Bio-Rad) according to the manufacturer’s instructions. The ddPCR reaction mixture (20 μl) contained 10 μl of a 2 × ddPCR Supermix (Bio-Rad), 1 μl of primers/probes each (500 nM of each primer and probe, S1 Table), and approximately 87.5 ng of cDNA. Droplets were generated using the Droplet Generator with 70 μl of Droplet Generator oil per well and 20 μl of every prepared sample in the DG8 cartridge (Bio-Rad). Droplets were dispensed into a 96 well PCR plate by aspirating 33 μl from the DG8 cartridge into each well. The PCR plate was then heat-sealed with a foil seal and placed in the thermocycler (C1000, Bio-Rad). Cycling consisted of 95°C for 10 min, followed by 40 cycles of 94°C for 30 s and 60°C for 1 min, one cycle of 98°C for 10 min, with a 4°C hold. The ramp rate was 2°C/sec. After the amplification, the droplets were read using the Droplet Reader for enumeration of the number of positive and negative droplets based on fluorescence. The number of template molecules per microliter of starting material was estimated by the QuantaSoft software using an internal Poisson algorithm. Although the use of reference genes is not considered mandatory in ddPCR assays, for time-course experiments it is strongly recommended [37]. The values for the gene of interest were normalized to the reference gene (*tubulin*) resulting in the relative quantity (RQ) of the gene of interest. Suitability of the *tubulin* as internal control gene for the investigated conditions was checked independently by the RT-qPCR procedure as described in [38]. In each ddPCR run the PCR reaction mixture without matrix was used as negative control (non template control, NTC).

Obtained ddPCR data were statistically analyzed by two-way analysis of variance (two-way ANOVA) with Box-Cox parametric power transformation. Fisher's least significant difference (LSD) method was used for multiple comparisons. Readings were considered significant when P was ˂0.05.

### Secretome extraction

About 100 ml of cultural broth samples were collected at specified times (3^rd^, 5^th^ and 8^th^ day) of fungal cultivation on GP, AL and BR media and filtered through syringe filters (0.45 μm, Sartorius, Germany) to remove small particles. Then, mycelium-free culture broth was concentrated 10-fold and simultaneously desalted by tangential flow ultrafiltration system Pellicon® XL with a 5 kDa Mw cut-off membrane Biomax 5 (Millipore, USA). Then the proteins were precipitated using Acetone-TCA method [39].

### Two-dimensional electrophoresis (2DE)

Two-dimensional electrophoresis was done according to O'Farrell [40] on a Protean II xi 2-D Cell system (Bio-Rad). 150–200 μg of the proteins were applied per a tube with Servalytes (pH gradient 3–10), and the isoelectrofocusing was performed using the following conditions: 100 V for 45 min, 200 V for 45 min, 300 V for 45 min, 400 V for 45 min, 500 V for 45 min, 600 V for 45 min, 700 V for 10 h, 900 V for 1.5 h.

After IEF, the samples were incubated at room temperature for 15 min in 0.5 M Tris-HCl buffer (pH 6.8) containing 6 M urea, 2% SDS, and 10 mM DTT (to prevent oxidation of sulfhydryl groups of the proteins). The subsequent electrophoresis of the samples was performed in a gradient SDS polyacrylamide gel (7.5–25%) at 300 V. The visualization of protein components in the gels was achieved by staining with AgNO_3_ or, for subsequent mass spectrometry of the protein samples, with Brilliant Blue R Staining Solution (Sigma, USA). Protein maps were analyzed using ImageMaster 2D Platinum v.7 program (GE Healthcare, UK).

### MALDI-TOF/TOF MS analysis

For MS analysis, small pieces of the gels with stained proteins of interest were cut out, incubated in 100 μl of 0.1 М NH_4_HCO_3_ containing 40% acetonitrile at 37°C for 20 min to remove coomassie brilliant blue, and the proteins were hydrolyzed by incubation at 37°C with 15 μg/ml solution of modified trypsin (Promega, USA) for 8 h. The solution above the gel containing the protein hydrolysate was collected and used for mass spectrometry with 2,5-dihydroxybenzoic acid (Aldrich, USA) as a matrix. Mass spectra were obtained using a Bruker Ultraflex II MALDI TOF/TOF mass spectrometer (Germany) with a reflectron.

Mass spectra data were processed using the FlexAnalysis 3.3 program (Bruker Daltonics, Germany). The search with combined data of the peptide masses and peptide fragmentation was performed by Biotools 3.2 (Bruker Daltonics). Ions score cut-off (p<0.05). Additionally, sequences of the peptides individually derived from the fragmentation data were analyzed using the *T*. *hirsuta* peroxidases dataset obtained after genome analysis.

## Results and discussion

### *Trametes hirsuta* PODs’ characteristics and phylogenetic analysis

**Fig 1:**
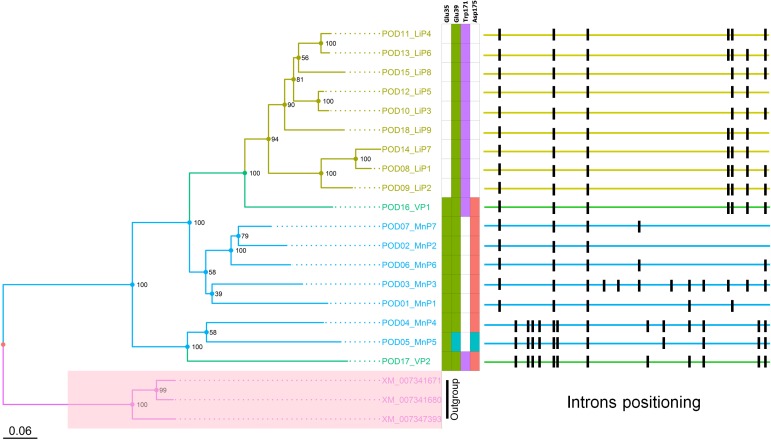
Maximum likelihood phylogenetic tree of *T*. *hirsuta* PODs, characteristic PODs amino acid residues and genes’ intron positions. Numbers at nodes are bootstrap percentages from 100 replicates. The outgroup containing the three peroxidase genes of *Auricularia subglabra* (XM_007341671.1, XM_007341680.1 and XM_007347393.1) is highlighted in pink. Characteristic LiP residues are highlighted in violet; characteristic MnP residues–in green and red. Non-standard aa residues are highlighted in blue. The numeration of aa residues is corresponding to *P*. *chrysosporium* PODs (MnP1 and LiP -H8) [37].

*In silico* analysis revealed 18 highly similar (Fig 1, S1 Fig) PODs genes in *T*. *hirsuta* genome (NCBI BioProject #PRJNA271118). The major contribution to the sequences difference is made by the regions with deletions/insertions rather than with amino acids residues replacements (S2 Fig). All genes of the PODs family contained several introns and possessed different exon-intron structure (Fig 1). Most splicing sites followed the GU-AG rule; however, we encountered several exceptions: GC-AG (POD2; third intron) and CG-AG (POD4; second intron), previously described in fungi [10].

Analysis of the locations of PODs within assembled contigs allowed us to define three gene clusters (S3 Fig). The first cluster (chromosome LIYB01000010) comprises POD 9, 18, 13, and 2, with 2000 bp average intergenic distance. The second cluster (chromosome LIYB01000011) comprises PODs 4, 10, 15, and 12, with average 15000 bp intergenic distance. The third cluster (chromosome LIYB01000011) comprises PODs 8 and 14, separated by 18212 bp. Other peroxidase genes were either located more than 60000 bp apart from other PODs or within separate contigs.

The existing classification of POD enzymes is based on the data on structural properties of protein molecules and the presence of characteristic amino acid residues (enzyme active center) [41,42]. We provisionally characterized all *T*. *hirsuta* PODs genes as "putative" according to presence or absence of typical amino acid residues: a tryptophan residue at the enzyme surface for LiPs, three residues located in an anionic pocket (two Glu residues and Asp), forming a Mn-binding site in MnPs, and both a catalytic tryptophan and a manganese-binding site for VPs [42] (Fig 1). Thus, we obtained seven putative MnPs (all of them are short MnPs), nine putative LiPs, and two putative VPs for *T*. *hirsuta*. Amino acid substitutions E39/Q39 and D175/Е175 are located in “characteristic” positions of MnP5. Similar “non-standard” residues were discovered previously, e.g., in MnP13 of *T*. *versicolor*, *Heterobasidion annosum*, etc. [9,43].

However, catalytic properties of the enzymes are not always consistent with the existing classification. For example, the peroxidase encoding gene in *P*. *ostreatus*, which was initially classified as a VP due to the presence of Trp-164 [44], was unable to oxidize high redox potential substrates directly and was defined as MnP [45]. At the same time, new LiP variant has been described for the white-rot fungus *Trametes cervina* [46], which lacks the LiP-characteristic catalytic Trp, possesses a solvent exposed tyrosyl residue (Tyr-181) as the oxidation site for high-redox potential substrates [5]. Thereby, a gene can only be referred to as a certain ligninolytic peroxidase after both amino acid sequence analysis and physical and chemical characteristic of the protein. It must also be supplemented with biochemical data on substrate specificity.

Phylogenetic analysis of the *T*. *hirsuta* PODs allowed us to determine three evolutionary related clades (Fig 1). Division of 18 POD genes discovered within the assembled genome into subgroups according to presence or absence of certain typical amino acids was well consistent with evolutionary relationships between genes in case of MnP and LiP: LiP form a single monophyletic clade within the tree; genes of putative MnP were divided into two clades, one of which has a common internal node with LiP clade. One of the isozymes assigned to VP is located within the LiP clade, while the second one belongs to the early diverged MnP clade. Thus, in our case, it will probably be more correct to define two different types of VP or to describe them as special subtypes of MnP and LiP. This case is apparently typical for the fungi of *Trametes* genus: similar division into monophyletic clades of MnP and LiP and allocation of VP genes are observed in *T*. *versicolor* as well [9,47]. Intron positions are generally consistent with phylogenetic relationships among the genes (Fig 1). It should be noted that genes that form the earliest diverged clade within the tree (including VP2) contain significantly greater number of introns than the rest. This is another confirmation of the different evolutionary faith of for VP1 and VP2 genes.

*In silico* analysis of the predicted amino acid sequences allowed to calculate the molecular mass, isoelectric point, and identify potential glycosylation sites in POD protein sequences of *T*. *hirsuta* (Table 1). All sequences contained a signal peptide typical for the secreted proteins. Predicted pI and Mw of sequences differed only slightly and were consistent with typical values for the PODs family [48]. The POD5 and POD17 proteins had greater number of potential glycosylation sites: both N-glycosylation (3 sites of per each protein) and О-glycosylation (19 and 14 sites respectively) (Table 1).

**Table 1:** Properties of the predicted protein sequences for *T*. *hirsuta* peroxidases. Genes with high expression rate/RQ (according to ddPCR data, discussed below) are marked in dark grey.

PODs	Predicted Isozymes	Predicted amino acid sequence		Number of potential O-glycosylation sites	Potential N-glycosylation sites
		pI	Mw, kDa		
POD1	MnP1	5.48	37.8	12	129 NLTA
POD2	MnP2	4.43	38.4	11	129 NISV
POD3	MnP3	4.70	38.4	9	129 NITA
POD4	MnP4	5.10	38.8	11	159 NVSR
					242 NGSN
POD5	MnP5	4.41	37.9	19	129 NVSF
					161 NDSQ
					237 NGTA
POD6	MnP6	4.82	38.7	13	128 NITT
POD7	MnP7	4.48	37.9	11	129 NITT
POD8	LiP1	5.10	39.8	8	287 NQTK
POD9	LiP2	4.73	39.4	9	287 NQTK
POD10	LiP3	4.72	39.3	8	129 NLSH
POD11	LiP4	4.63	38.6	7	129 NLSH
POD12	LiP5	4.63	38.6	8	129 NLSH
POD13	LiP6	4.89	39.6	7	129 NLSH
POD14	LiP7	4.86	39.2	9	287 NQTK
POD15	LiP8	4.55	39.3	8	129 NLSV
POD16	VP1	4.89	39.6	9	129 NLSH
POD17	VP2	4.52	38.8	14	127 NVSF
					159 NDSQ
					235 NGTL
POD18	LiP9	4.41	38.3	14	129 NLSN
					240 NGTT

### Differential transcription of peroxidase genes. Effect of dye and lignin on the peroxidases genes expression

Existing data on expression of genes encoding ligninolytic enzymes suggest that production of lignin-modifying enzymes is time-dependent and can be related to the stage of wood degradation [49–51]. This suggests turning on the expression of additional isozymes, depending on accumulation of the specific inducers on different stages of lignin degradation [52].

We traced the expression dynamics of individual isozymes of PODs on various stages of fungal growth and studied the influence of two inducers: bromocresol green as a simple model compound containing three phenol residues, and branched alkali lignin as a more complex compound. The presence of free phenolic moiety in sulfonphthalein dye and alkali lignin makes them suitable substrates as well as substrate-specific regulators for peroxidases associated with lignin degradation in fungi [53,54].

**Fig 2:**
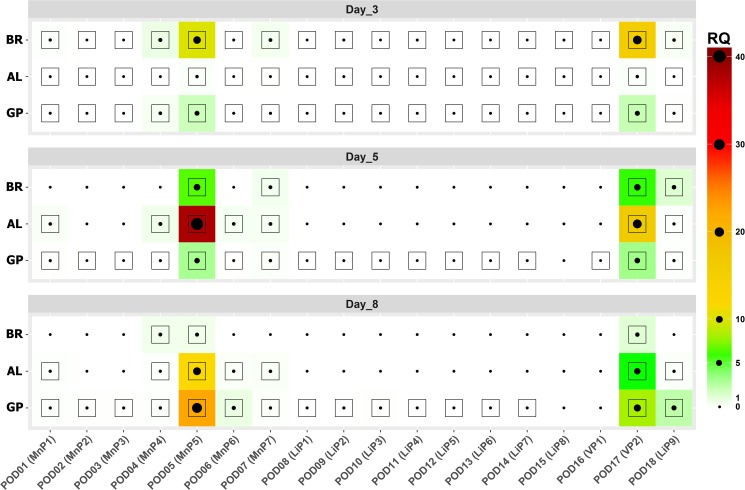
Relative expression of PODs genes under different culture conditions. Gene wise expression of PODs. Each doted square represents absolute expression normalized on internal control gene expression (RQ). The expression correlates both with the size of a dot and the color of a box. Samples in which expression was not detected depicted as small unsquared dots. GP, BR and AL stand for glucose-peptone medium and glucose-peptone medium with addition of bromocresol green and alkali lignin respectively.

**Fig 3:**
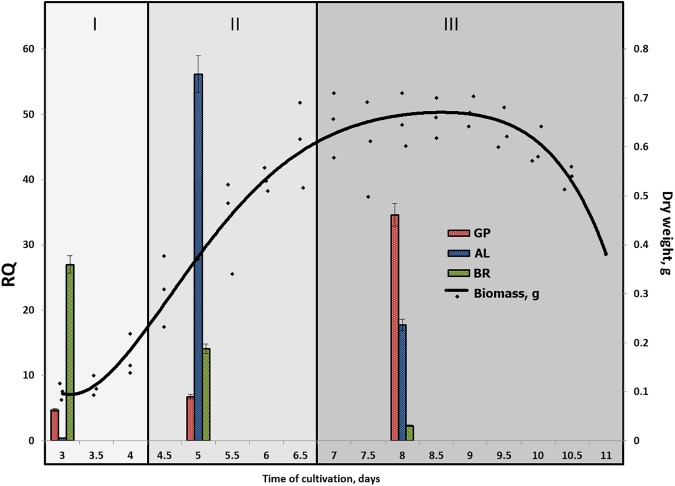
Total relative expression of PODs complex at different growth stages and under different culture conditions of *T*. *hirsuta*. I, lag phase; II, exponential growth phase; III, stationary and lysis phase. The samples were taken in 12 h interval. Each dot represents arithmetic mean from three biological replicates on each cultivation media (GP, BR and AL). Polynomial regression line is superimposed on the mean scatter plot. Error bars represent standard deviations.

The expression profiles of peroxidase genes under submerged cultivations were analyzed by ddPCR (Figs 2 and 3, S2 Table). Gene wise expression dynamics of PODs were shown to differ significantly depending on the medium and time of cultivation.

In the case of GP medium the transcription levels (RQ value, see MM section) of only two isozymes POD5 and POD17 were greater than 1 at all growth stages, with a gradual increase by day 8. However, the most significant change in the transcriptional level depending on growth phase was marked for low expressed isozyme POD18—an increase of 30 times during the transition from exponential growth phase (5th day) to stationary phase (8th day) (RQ– 0.07 and 2.39 respectively). Under these conditions, generally isozymes demonstrate the increase of transcription level from stage to stage. The transcription decrease is observed in POD4, while for POD15 and POD16 the absolute zero of expression is detected at stationary phase of growth.

In the case of AL medium, RQ for all isozymes was less than 1 at the 3rd day. POD5 and POD17 showed a significant increase in the transcription level on the day 5 (RQ—38 and 16 respectively), and about 3-fold recession at the day 8 (RQ—11 and 5, respectively). Lack of expression was observed for 11 isozymes already at 5th day.

On BR medium at the 3th day of cultivation the RQ value greater than 1 was detected only for POD5 and POD17 (RQ—11 and 15 respectively) with subsequent gradual decrease in the transcription of most genes up to the day 8. On the 8th day RQ value of POD5 was even less than 1. However, two isozymes showed different dynamics in the expression levels: POD4 isozyme was well expressed during the cultivation, but totally lost on the 5^th^ day, and POD18 had the highest level of its expression on day 5^th^ day and disappeared at the end of cultivation (on the day 8).

Since ddPCR is an absolute quantification method, it allows to assess phase- and inducer-dependent PODs complex expression alterations and contribution of each isozyme into the cumulative work of peroxidase complex.

POD5 (putative MnP5) and POD17 (putative VP2) were the predominant transcripts during all cultivation time and on all studied media (Fig 2). On GP medium, these genes made up 88% (42% POD5 and 46% POD17), 94% (45% POD5 and 49% POD17), and 89% (65% POD5 and 24% POD17) of total POD transcripts expression on days 3, 5, and 8, respectively. On AL medium, these genes made up 80% (41% POD5 and 39% POD17), 98% (69% POD5 and 29% POD17), and 80% (63% POD5 and 32% POD17) of total POD transcripts on days 3, 5, and 8, respectively. On BR medium, these genes made up 94% (37% POD5 and 57% POD17), 87% (45% POD5 and 42% POD17), and 77% (25% POD5 and 52% POD17) of total POD transcripts on days 3, 5, and 8, respectively.

Biologically significant expression of all peroxidase genes could be observed on all studied media on the day 3 of cultivation (Fig 2). At this time point total expression of peroxidases on AL medium was 11.5 times lower and on BR medium 5.8 times higher comparing to GP medium (Fig 3). Further dynamics of changes in expression levels (days 5 and 8) are considerably different in all three cases (Figs 2 and 3).

Gradual increase in total expression of peroxidase genes was observed on GP medium: 1.5-fold on day 5 and 7.5-fold on day 8, relative to initial expression levels (day 3) (Fig 3). Changes to the spectrum of expressed peroxidases were insignificant (Fig 2). POD4 must be noted among the genes with low expression levels; its transcripts made up 7.5% of total POD transcripts on day 3, and POD18, which comprised 6.8% of total POD transcripts on day 8. Contribution of other genes on all media during the whole studied period was less than 2.5% for each gene.

AL medium is characterized by rapid increase of PODs expression on day 5 (140 times higher compared to day 3) and its subsequent decrease on day 8 (Fig 3). Expression of all peroxidase genes except two main ones was less than 1.5% of total POD transcripts.

The decrease of total level of expression was observed on BR medium: 2 times lower on day 5 and 11.7 times lower on day 8, relative to initial expression level (day 3) (Fig 3). Here, similar to AL medium, the spectrum of peroxidases was rapidly reduced (Fig 2), only four peroxidases remaining by day 5 (POD 5, 7, 17, and 18); contribution of two genes with low expression levels was 13%. By day 8, expression of highly expressed PODs genes remained, while the spectrum of genes with low expression changed: POD4 was expressed (22.3% of total POD transcripts) instead of POD9 and POD18.

Thus, the highest expression level on all studied media is noted for two genes, POD5 and POD17, which are not united in a single gene cluster, but belong to the same phylogenetic clade. Expression of the third gene from this clade, POD4, was considerably lower compared to these two genes, but higher than for other peroxidases (Fig 2). Also moderate expression was observed for POD18. Portion of the last two genes increased during late stages of cultivation, which may be connected to participation of these peroxidases in utilization of accumulated toxic metabolic products. Increased expression of POD5 and POD17 genes, as well as POD4 on medium with the dye, was already observed on day 3, and on day 5 on the medium supplied with alkali lignin. Expression of manganese peroxidases POD5 (MnP5) and POD4 (MnP4) was more effective in response to alkali lignin introduction (2.5 to 3-fold), while the dye increased the expression of versatile peroxidase POD17 (VP2). These data is consistent with the previous finding that the increase in the degree of halogenation of dyes made these substrates more suitable for VP [55].

Generally, the dynamics of peroxidase expression during the growth of *T*. *hirsuta* under three different culture conditions (control medium, complex biopolymer, and low-molecular-weight aromatic compound) can be explained on the basis of general principles of ecological physiology.

On the third day of cultivation culture broth contained only 10–20% of initial carbohydrate content. This data is in agreement with [25,56,57]. So the main source of carbon in the medium (glucose) was almost depleted, and fungus gets under the conditions of carbon starvation and is “searching” for the sources of carbon. In the environment conditions cellulose is hidden from the direct access of cellulolytic enzymes by a lignin, and the fungus begins to express most genes of ligninolytic complex [51]. Expression of the whole complex of peroxidases on the day 3 can be described as a “search” for the shielded sources of carbon in an ambient environment. For example, a gradual increase in expression of the whole complex of peroxidases, up to the stationary phase, is observed during the growth on control medium. During the growth on alkali lignin, however, cleavage of low-molecular-weight phenol components, which apparently signal back to the fungus, changes the expression profile by excluding a number of peroxidases and dramatically increasing the expression of individual genes. Similar situation is observed during growth with the introduction of synthetic dye. Presence of the effectors in cultural broth stimulates expression of just several genes simultaneously excluding expression of all others.

Considering the lack of significant fluctuations in growth curve on all three media, we assumed that the main signals of expression of certain peroxidases can be: the available carbon and nitrogen in the medium, or the presence and concentration of specific low-molecular-weight compounds, possibly of phenolic nature (lignin derivative). This is confirmed by the presence of multiple XRE and CRE elements in promoter regions of PODs genes [10]. Inducers described in the present study possessed typical features of lignin structural units: location of substitutes within the molecules, specific for syringyl phenylpropanoid units of lignin. In hardwood trees, which are typically inhabited by *T*. *hirsuta*, lignin is comprised mainly by two types of monomers: guaiacyl (G-subunits) and syringyl (S-subunits) and portion of the latter approximates 65%. MnP was previously demonstrated to be able to participate in Сα oxidation of β–1 syringyl model structures of lignin [48,58]. Apparently, supplying the cultivation medium with compounds of phenolic nature (aromatic structures) can induce expression of these enzymes [59]. It can be suggested that transcription of POD5, POD17, and POD4 genes is positively regulated by the compounds of phenolic nature with typical syringyl structure. The structure of monomeric units of lignin and the dye are identical; however, induction (and expression) of POD on BR medium begins earlier than in medium with lignin, apparently due to the existence of an initial stage regulating expression of peroxidases.

### Analysis of extracellular peroxidases profiles

MALDI TOF/TOF MS demonstrated that POD5 and POD17 were the main genes secreted by the basidiomycete under the given conditions of the study (see MM section, S3 and S4 Tables). In addition, POD7 was found during the whole process of cultivation and on all media. Presence of the following peroxidases was also demonstrated in cultural broth: POD2, POD3, POD6, and POD18 (S4 Table). POD5 and POD17 were produced as multiple isoforms (up to 14 isoforms of POD5, up to six isoforms of POD17), and their number altered during cultivation. Other underrepresented peroxidases were typically produced as a single isoform during 5 to 8 days of cultivation. It should be noted that highly expressed peroxidases (POD5 and POD17) of *T*. *hirsuta* are characterized by greater number of potential sites of glycosylation (Table 1), which may be the cause of multiple protein isoforms formation.

The fact that, despite similar levels of expression of PODs (by day 3 of cultivation), a limited set of enzymes is secreted in cultural broth, is surprising. In the study [12], the absence of one of the MnPs isoforms from the secretome (whilst presence in intracellular proteome) of the *P*. *ostreatus* lignocellulose cultures was related to impaired secretion of the protein. This can also be related to the glycolysis degree and to extracellular proteases resistance [52].

Although *T*. *hirsuta* possess nine putative LiP genes, we have detected neither significant expression level nor significant protein production of these isozymes during cultivation. With the exception of POD18 (LiP9), which expression was detected on the day 5 for BR and on 8^th^ for AL media and production appeared to be only on day 8. Earlier for *P*. *ostreatus*, a lack of LiP genes in lignin-degrading system was shown. It was suggested that the role generally played by LiP has been assumed by VP in some Agaricales [45]; it is also likely to be possible for Polyporales. LiP is possibly more substrate-specific or necessary for detoxification of the medium inhabited by the fungus.

Patterns of expression and production of peroxidase genes on media with bromocresol and lignin are similar in their nature but differ in time–for AL the peak of expression lags 2 days of cultivation. Processes of degradation of lignocellulose materials and decomposition of xenobiotics by basidiomycetes can include at the first stage production of highly reactive low-molecular-weight compounds, functioning as oxidants,. These compounds specifically “process” wood and facilitate the accessibility of lignin for an enzyme attack. Hydroxyl radicals (ОН*) play the main role in these processes. ОН* radicals attack lignocellulose material and/or polysaccharides of the cell wall, causing decomposition of biopolymers and facilitating the entry of lignin-modifying enzymes [48]. The byproducts of these processes may serve as signals to drive the expression of peroxidase genes and specific proteins production.

Based on previous data [48,51,60] and data obtained in this study, the main route of substrate degradation in *T*. *hirsuta*, which includes peroxidase complex, is apparently an enzyme-mediated route that includes production of radicals and initiation of chain enzyme reactions. Manganese POD5 (MnP5) as well as versatile POD17 (VP2) peroxidases acts the first. VPs considered being enzymes with high substrate specificity, capable of both directed and mediated conversion of phenols, dyes, and lignin without mediators necessary for LiP and MnP [5]. Then, by days 8–10, additionally POD6, POD2, POD3, and POD18 are produced, which are apparently capable of selective oxidation of bonds in simple phenol compounds. The role of POD7 is yet unclear; only one isoform is present on the secretome maps, which remains on the same level throughout the whole studied period (up to day 8 at least).

Changes in the composition of secretome depending on the medium of cultivation are typical for *T*. *hirsuta*. For example, we observed dramatic alterations after supplying the synthetic medium with lignocellulose substrate [51]. These alterations were associated with the global secretome profiles as well as with peroxidase complex produced by the fungus. Based on data obtained we can propose that the vital PODs functions are supported by universal constitutively expressed isozymes while the subtle regulation and a specific response to changing life conditions are implemented by set of low expressed isozymes. On the other hand subtle regulation and a specific response to changing life conditions is implemented by set of low expressed isozymes. Here we have shown that various peroxidases are apparently differing in specific roles, providing plasticity and vital efficiency of the fungal species under any growth conditions. Moreover the comparison of data on expression of peroxidase genes and their production, led to the question on the effectiveness of the secretion process of peroxidases in fungi.

## Supporting information

S1 Fig PODs identity tables. (PDF)

S2 Fig PODs Protein alignment. (TIF)

S3 Fig Gene clusters. (TIF)

S1 Table Primers and TaqMan probes for PODs. (PDF)

S2 Table Numerical ddPCR data. (PDF)

S3 Table Identified secreted PODs. (PDF)

S4 Table Secreted PODs during cultivation on different media. (PDF)
