# Supplementary material for: Lignin-degrading peroxidases in white-rot fungus Trametes hirsuta 072. Absolute expression quantification of full multigene family
Source: PLoS One. 2017 Mar 16;12(3):e0173813. doi: 10.1371/journal.pone.0173813 (PMC5354401; doi:10.1371/journal.pone.0173813)
Supplement: S1 Fig — (PDF) [file pone.0173813.s001.pdf]

S1 Fig. ***Trametes hirsuta*** **072 Lignin-degrading peroxidases identity tables.**

% Identity Proteins

	POD1 (MnP1)	POD2 (MnP2)	POD3 (MnP3)	POD4 (MnP4)	POD5 (MnP5)	POD6 (MnP6)	POD7 (MnP7)	POD8 (LiP1)	POD9 (LiP2)	POD10 (LiP3)	POD11 (LiP4)	POD12 (LiP5)	POD13 (LiP6)	POD14 (LiP7)	POD15 (LiP8)	POD16 (VP1)	POD17 (VP2)	POD18 (LiP9)
POD1 (MnP1)		68	70	57	54	70	73	60	60	60	61	62	62	59	60	62	48	60
POD2 (MnP2)	68		75	53	53	82	87	62	63	66	67	67	67	61	67	67	51	64
POD3 (MnP3)	70	75		55	54	74	75	60	62	64	65	65	64	59	65	65	51	62
POD4 (MnP4)	57	53	55		65	55	55	51	53	49	49	50	49	53	51	51	57	51
POD5 (MnP5)	54	53	54	65		52	54	48	50	49	48	50	47	49	50	49	61	50
POD6 (MnP6)	70	82	74	55	52		84	64	63	66	66	67	66	62	65	65	51	65
POD7 (MnP7)	73	87	75	55	54	84		64	66	68	68	70	69	62	69	67	51	65
POD8 (LiP1)	60	62	60	51	48	64	64		85	75	75	76	75	93	74	66	49	75
POD9 (LiP2)	60	63	62	53	50	63	66	85		77	77	78	77	87	76	70	51	76
POD10 (LiP3)	60	66	64	49	49	66	68	75	77		89	97	89	74	86	74	49	83
POD11 (LiP4)	61	67	65	49	48	66	68	75	77	89		89	97	73	87	76	48	82
POD12 (LiP5)	62	67	65	50	50	67	70	76	78	97	89		89	74	86	75	50	85
POD13 (LiP6)	62	67	64	49	47	66	69	75	77	89	97	89		73	87	75	48	83
POD14 (LiP7)	59	61	59	53	49	62	62	93	87	74	73	74	73		72	66	49	74
POD15 (LiP8)	60	67	65	51	50	65	69	74	76	86	87	86	87	72		73	49	81
POD16 (VP1)	62	67	65	51	49	65	67	66	70	74	76	75	75	66	73		51	70
POD17 (VP2)	48	51	51	57	61	51	51	49	51	49	48	50	48	49	49	51		49
POD18 (LiP9)	60	64	62	51	50	65	65	75	76	83	82	85	83	74	81	70	49	

% Identity Nucleotides

	POD1 (MnP1)	POD2 (MnP2)	POD3 (MnP3)	POD4 (MnP4)	POD5 (MnP5)	POD6 (MnP6)	POD7 (MnP7)	POD8 (LiP1)	POD9 (LiP2)	POD10 (LiP3)	POD11 (LiP4)	POD12 (LiP5)	POD13 (LiP6)	POD14 (LiP7)	POD15 (LiP8)	POD16 (VP1)	POD17 (VP2)	POD18 (LiP9)
POD1 (MnP1)		68	69	61	58	68	71	64	63	65	66	66	64	64	65	65	59	65
POD2 (MnP2)	68		72	59	57	75	80	65	66	66	67	69	67	65	68	67	59	67
POD3 (MnP3)	69	72		60	58	72	72	65	65	67	67	68	66	63	68	67	57	66
POD4 (MnP4)	61	59	60		64	58	59	59	59	57	57	58	56	58	58	58	64	56
POD5 (MnP5)	58	57	58	64		57	56	55	55	56	54	57	53	53	56	55	59	54
POD6 (MnP6)	68	75	72	58	57		79	66	65	68	67	69	69	64	68	67	58	66
POD7 (MnP7)	71	80	72	59	56	79		66	67	68	69	70	69	65	70	68	58	68
POD8 (LiP1)	64	65	65	59	55	66	66		78	75	73	76	73	90	74	69	59	72
POD9 (LiP2)	63	66	65	59	55	65	67	78		72	74	74	74	78	74	69	58	72
POD10 (LiP3)	65	66	67	57	56	68	68	75	72		81	95	81	74	81	74	58	76
POD11 (LiP4)	66	67	67	57	54	67	69	73	74	81		81	87	73	81	74	59	76
POD12 (LiP5)	66	69	68	58	57	69	70	76	74	95	81		80	75	80	75	59	77
POD13 (LiP6)	64	67	66	56	53	69	69	73	74	81	87	80		73	81	74	59	77
POD14 (LiP7)	64	65	63	58	53	64	65	90	78	74	73	75	73		72	68	59	72
POD15 (LiP8)	65	68	68	58	56	68	70	74	74	81	81	80	81	72		73	60	74
POD16 (VP1)	65	67	67	58	55	67	68	69	69	74	74	75	74	68	73		60	70
POD17 (VP2)	59	59	57	64	59	58	58	59	58	58	59	59	59	59	60	60		56
POD18 (LiP9)	65	67	66	56	54	66	68	72	72	76	76	77	77	72	74	70	56	
