# Supplementary figures and images for: Lignin-degrading peroxidases in white-rot fungus Trametes hirsuta 072. Absolute expression quantification of full multigene family
Source: PLoS One. 2017 Mar 16;12(3):e0173813. doi: 10.1371/journal.pone.0173813 (PMC5354401; doi:10.1371/journal.pone.0173813)

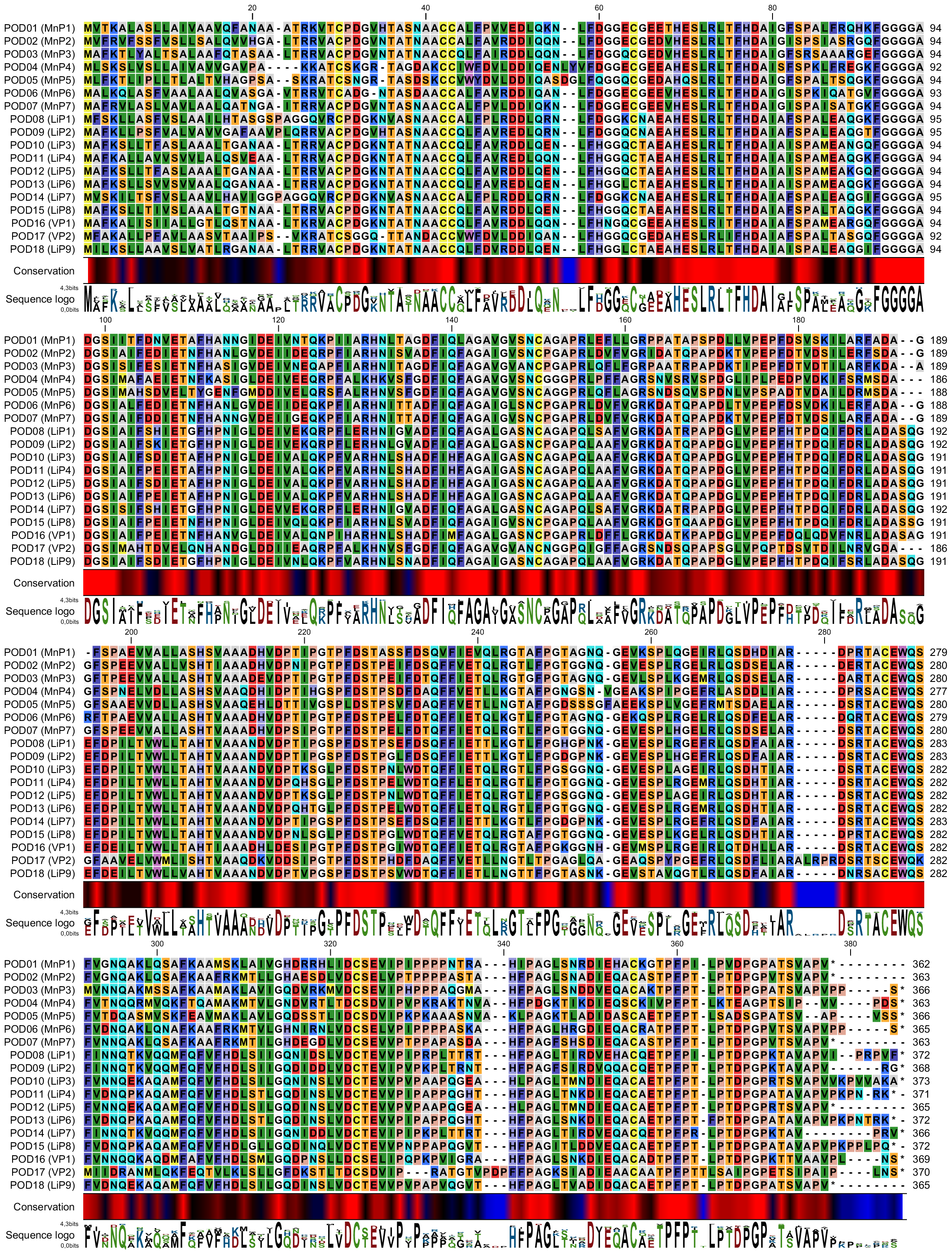

Supplement: S2 Fig — (TIF) [file pone.0173813.s002.tif]

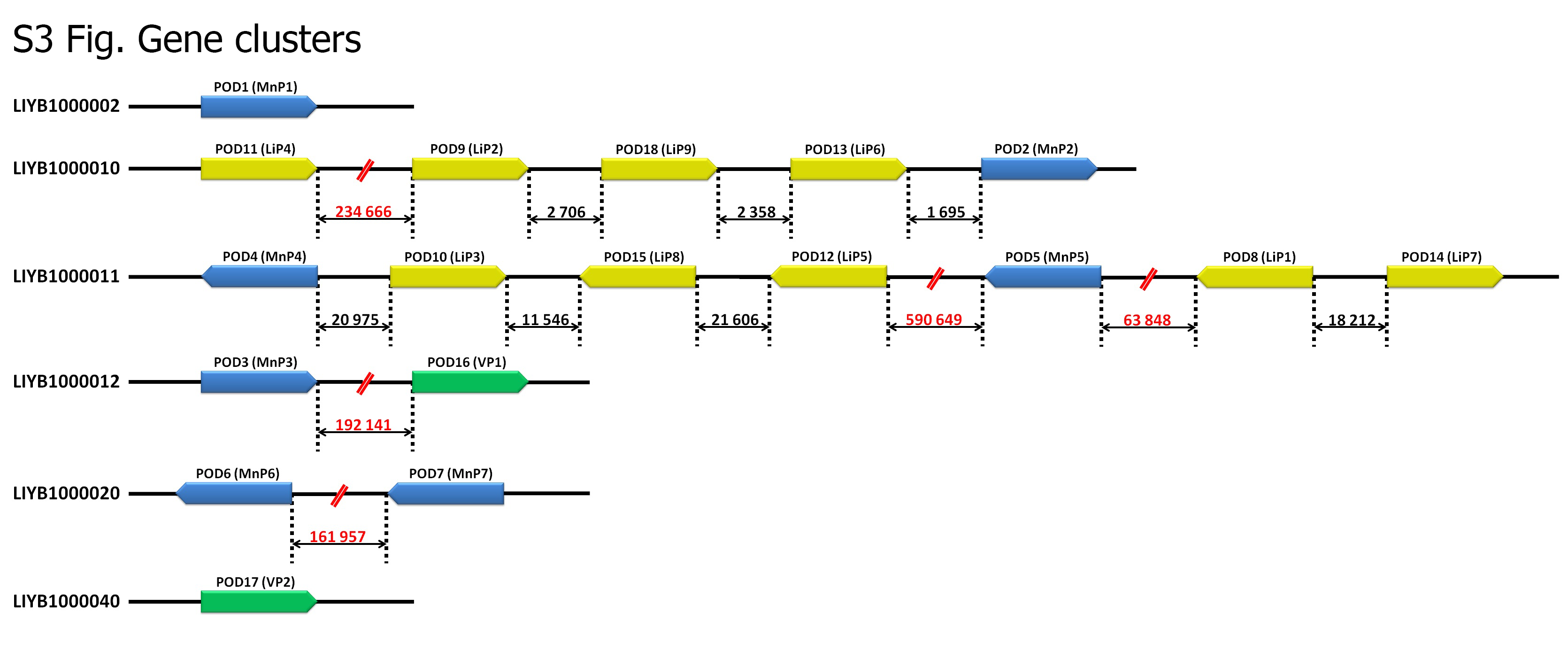

Supplement: S3 Fig — (TIF) [file pone.0173813.s003.tif]
