# Supplementary material for: Lignin-degrading peroxidases in white-rot fungus Trametes hirsuta 072. Absolute expression quantification of full multigene family
Source: PLoS One. 2017 Mar 16;12(3):e0173813. doi: 10.1371/journal.pone.0173813 (PMC5354401; doi:10.1371/journal.pone.0173813)
Supplement: S1 Table — (PDF) [file pone.0173813.s004.pdf]

S1 Table. Primers and TaqMan probes.

Gene	Primer Name	5'Reporter/Primer Sequence/3'Quencher
**POD1 (MnP1)**	POD1_For	5'-AGTCTCTCCGCCTCACAT-3'
	POD1_Rev	AACGTGATGATGGAGCCG-3'
	POD1_TaqMan	[5']Fam-CCCTGCCCTCTTTCGCCAGCACA-BHQ1[3']
**POD2 (MnP2)**	POD2_For	5'-CATTGCCCGTCACAACAT-3'
	POD2_Rev	5'-AGGATGCTATCGACGGTG-3'
	POD2_TaqMan	[5']R6G-TCGTCTTGATGTCTTTGTTGGACGCATAG-BHQ2[3']
**POD3 (MnP3)**	POD3_For	5'-AGACACTCTACGCCCTCAC-3'
	POD3_Rev	5'-GTTCGTCGCGGTGTTCACG-3'
	POD3_TaqMan	[5']Fam-AACCGCTAGCGCCGCACTCACTCGTC-BHQ1[3']
**POD4 (MnP4)**	POD4_For	5'-AGAAGCTAAGTCCCCCATCC-3'
	POD4_Rev	5'-GCTGCTGGTTGGTGACGAA-3'
	POD4_TaqMan	[5']Fam-CCTAGCGATGAGGTCGTCCGAGG-BHQ1[3']
**POD5 (MnP5)**	POD5_For	5'-GGCTTTGCAGAGGAGAAGT-3'
	POD5_Rev	5'-TGACCATCGAAGCCTGGTC-3'
	POD5_TaqMan	[5']Fam-TCAGATGCCGAACTCGCCAGG-BHQ1[3']
**POD6 (MnP6)**	POD6_For	5'-GGAGGTGCATGAGTCCCTC-3'
	POD6_Rev	5'-CGTGGAAGTTGGTCTCGAT-3'
	POD6_TaqMan	[5']R6G-GCCCAAGATCCAGGCTACGGGCG-BHQ2[3']
**POD7 (MnP7)**	POD7_For	5'-CGTCTCTTGTAGCGGTTCTT-3'
	POD7_Rev	5'-GCGTTGGAGGCGGTGTTAA-3'
	POD7_TaqMan	[5']R6G-CAGGCTACCAACGGTGCTATTACTCGTCG-BHQ2[3']
**POD8 (LiP1)**	POD8_For	5'-GTTGGCTTCCTTCGTCTCTC-3'
	POD8/14_Rev	5'-TTGCTCGCGACGTTCTTG-3'
	POD8_TaqMan	[5']Fam-CAGCGGAAGTCCAGCGGGGGGCCAA-BHQ1[3']
**POD9 (LiP2)**	POD9_For	5'-TTGCTCCCTTCCTTCGTCG-3'
	POD9_Rev	5'-AGCACGCTGCATTGGAAG-3'
	POD9_TaqMan	[5']R6G-TGCTGCTGTGCCGCTTCAGCGCCGT-BHQ2[3']
**POD10 (LiP3)**	POD10_For	5'-GAACGCCGCGCTCACC-3'
	POD10/12_Rev	5'-AAGTTCTCCTGGAGGTCGT-3'
	POD10_TaqMan	[5']Fam-TGGAAAGAACACGGCGACGAACGCCGCG-BHQ1[3']
**POD11 (LiP4)**	POD11_For	5'-CAAGTTTGGTGGTGGAGGTG-3'
	POD11_Rev	5'-GTTCGAAGCACCGATGGCA-3'
	POD11_TaqMan	[5']Fam-ACGCCATAACCTCTCGCACGCT-BHQ1[3']
**POD12 (LiP5)**	POD12_For	5'-CTCACTCGGCGCGTCG-3'
	POD10/12_Rev	5'-AAGTTCTCCTGGAGGTCGT-3'
	POD12_TaqMan	[5']R6G-CGGCAAGAATACCGCAACCAACGCCGCA-BHQ2[3']
**POD13 (LiP6)**	POD13_For	5'-CTCTCTGTTGTCTCCGTTGT-3'
	POD13_Rev	5'-GTTGGTCGCGGTGTTCTTG-3'
	POD13_TaqMan	[5']Fam-CGCGAATGCGGCGTTGACCCGC-BHQ1[3']
**POD14 (LiP7)**	POD14_For	5'-CCTCCTTTGTCTCTCTCGCC-3'
	POD8/14_Rev	5'-TTGCTCGCGACGTTCTTG-3'
	POD14_TaqMan	[5']R6G-CATCGGAGGTCCCGCTGGCGGCCAA-BHQ2[3']
**POD15 (LiP8)**	POD15_For	5'-TATCGCGTTCTCTCCCGC-3'
	POD15_Rev	5'-CCAGGGCAGTTCGAGACG-3'
	POD15_TaqMan	[5']R6G-CCAGAGATTGAGACAAACTTCCACCCCAA-BHQ2[3']

**POD16 (VP1)**	POD16_For	5'-TCTCGCATGCCGACTTCA-3'
	POD16_Rev	5'-CGGTTGAAGACGTCTTGGAG-3'
	POD16_TaqMan	[5']Fam-CCGCCTCGACTTCTTCCTCGGCCGC-BHQ1[3']
**POD17 (VP2)**	POD17_For	5'-GCGAGGCCCAATCCCCTTA-3'
	POD17_Rev	5'-CGGTCTGCTCGAACTTCTG-3'
	POD17_TaqMan	[5']R6G-CTCCAGTCAGACTTCCTCATTGCTCGC-BHQ2[3']
**POD18 (LiP9)**	POD9_For	5'-TTGCTCCCTTCCTTCGTCG-3'
	POD9_Rev	5'-AGCACGCTGCATTGGAAG-3'
	POD9_TaqMan	[5']Fam-CTCCCAAGGAGAGTTCGACGAGATCCT-BHQ1[3']
**Tub**	Tub Fwd	5'-ACACTCAAGCTGACAACA-3'
	Tub Rev	5'-ATGAAGAAGTGGAGACGA-3'
	Tub_TaqMan	[5']Fam-ACCAGGGAAACGCAAGCAA-BHQ1[3']
