# Supplementary material for: Lignin-degrading peroxidases in white-rot fungus Trametes hirsuta 072. Absolute expression quantification of full multigene family
Source: PLoS One. 2017 Mar 16;12(3):e0173813. doi: 10.1371/journal.pone.0173813 (PMC5354401; doi:10.1371/journal.pone.0173813)
Supplement: S2 Table — (PDF) [file pone.0173813.s005.pdf]

S2 Table. Numerical ddPCR data.

GP-medium

	copy/µl			gene copy/tubulin copy				Percentage		
	Day 3	Day 5	Day 8	Day 3	Day 5	Day 8		Day 3	Day 5	Day 8
POD1 (MnP1)	6,81E+03	6,92E+03	2,99E+03	2,92E-02	4,61E-02	6,48E-02		0,62	0,68	0,19
POD2 (MnP2)	1,44E+03	1,33E+02	2,04E+03	6,15E-03	8,87E-04	4,43E-02		0,13	0,01	0,13
POD3 (MnP3)	1,55E+03	3,79E+02	2,82E+03	6,62E-03	2,53E-03	6,12E-02		0,14	0,04	0,18
POD4 (MnP4)	8,07E+04	6,29E+03	5,88E+03	3,46E-01	4,19E-02	1,28E-01		7,41	0,62	0,37
POD5 (MnP5)	4,54E+05	4,59E+05	1,04E+06	1,94E+00	3,06E+00	2,26E+01		41,63	45,30	65,17
POD6 (MnP6)	5,27E+03	3,08E+03	3,68E+04	2,26E-02	2,05E-02	8,00E-01		0,48	0,30	2,31
POD7 (MnP7)	9,31E+03	2,61E+04	6,12E+03	3,99E-02	1,74E-01	1,33E-01		0,85	2,57	0,38
POD8 (LiP1)	1,30E+03	2,00E+02	1,57E+03	5,55E-03	1,33E-03	3,41E-02		0,12	0,02	0,10
POD9 (LiP2)	2,05E+03	8,11E+02	1,82E+03	8,80E-03	5,41E-03	3,94E-02		0,19	0,08	0,11
POD10 (LiP3)	2,49E+03	2,93E+02	2,69E+03	1,07E-02	1,95E-03	5,85E-02		0,23	0,03	0,17
POD11 (LiP4)	1,06E+03	2,39E+02	1,29E+03	4,54E-03	1,60E-03	2,80E-02		0,10	0,02	0,08
POD12 (LiP5)	1,08E+03	2,19E+02	1,22E+03	4,64E-03	1,46E-03	2,66E-02		0,10	0,02	0,08
POD13 (LiP6)	1,59E+03	4,99E+02	1,43E+03	6,80E-03	3,32E-03	3,09E-02		0,15	0,05	0,09
POD14 (LiP7)	1,47E+03	6,45E+02	1,40E+03	6,28E-03	4,30E-03	3,04E-02		0,13	0,06	0,09
POD15 (LiP8)	4,37E+02	0	0	1,87E-03	0	0		0,04	0	0
POD18 (LiP9)	1,17E+04	1,60E+03	2,23E+05	6,00E-02	7,16E-02	2,39E+00		1,29	1,06	6,89
POD16 (VP1)	1,02E+02	1,33E+02	0	4,36E-04	8,87E-04	0		0,01	0,01	0
POD17 (VP2)	5,06E+05	4,97E+05	3,77E+05	2,17E+00	3,32E+00	8,19E+00		46,38	49,11	23,67
TUB (first set)	2,33E+05	1,50E+05	4,61E+04				Total	100,00	100,00	100,00

“AL” samples

	copy/µl			gene copy/tubulin copy				Percentage		
	Day 3	Day 5	Day 8	Day 3	Day 5	Day 8		Day 3	Day 5	Day 8
POD1 (MnP1)	4,35E+02	2,97E+02	1,70E+01	1,16E-02	2,55E-01	7,63E-02		2,75	0,45	0,43
POD2 (MnP2)	3,64E+01	0	0	9,72E-04	0	0		0,23	0	0
POD3 (MnP3)	7,14E+01	0	0	1,91E-03	0	0		0,45	0	0
POD4 (MnP4)	2,65E+02	6,93E+02	1,62E+01	7,08E-03	5,96E-01	7,25E-02		1,68	1,06	0,41
POD5 (MnP5)	6,53E+03	4,43E+04	2,51E+03	1,74E-01	3,81E+01	1,13E+01		41,30	67,91	63,35
POD6 (MnP6)	5,44E+01	4,08E+02	4,76E+01	1,45E-03	3,50E-01	2,14E-01		0,34	0,62	1,20
POD7 (MnP7)	6,90E+02	4,46E+02	5,61E+01	1,84E-02	3,83E-01	2,52E-01		4,37	0,68	1,42
POD8 (LiP1)	1,68E+02	0	0	4,50E-03	0	0		1,06	0	0
POD9 (LiP2)	2,47E+02	0	0	6,58E-03	0	0		1,56	0	0
POD10 (LiP3)	7,58E+01	0	0	2,03E-03	0	0		0,48	0	0
POD11 (LiP4)	5,78E+01	0	0	1,54E-03	0	0		0,37	0	0
POD12 (LiP5)	6,36E+01	0	0	1,70E-03	0	0		0,40	0	0
POD13 (LiP6)	1,46E+02	0	0	3,91E-03	0	0		0,92	0	0
POD14 (LiP7)	3,18E+02	0	0	8,49E-03	0	0		2,01	0	0
POD15 (LiP8)	2,74E+02	0	0	7,31E-03	0	0		1,73	0	0
POD18 (LiP9)	5,44E+01	2,23E+02	3,43E+01	1,45E-03	1,10E-01	1,54E-01		0,34	0,20	0,87
POD16 (VP1)	8,16E+01	0	0	2,18E-03	0	0		0,52	0	0
POD17 (VP2)	6,24E+03	1,90E+04	1,28E+03	1,67E-01	1,63E+01	5,74E+00		39,47	29,07	32,32
TUB (first set)	3,74E+04	1,16E+03	2,23E+02				Total	100,00	100,00	100,00

“BR” sample

	copy/µl			gene copy/tubulin copy				Percentage		
	Day 3	Day 5	Day 8	Day 3	Day 5	Day 8		Day 3	Day 5	Day 8
POD1 (MnP1)	5,90E+03	0	0	7,78E-02	0	0		0,29	0	0
POD2 (MnP2)	1,08E+02	0	0	1,43E-03	0	0		0,01	0	0
POD3 (MnP3)	2,91E+01	0	0	3,84E-04	0	0		0,00	0	0
POD4 (MnP4)	5,66E+04	0	7,26E+01	7,47E-01	0	5,08E-01		2,77	0	22,30
POD5 (MnP5)	7,60E+05	1,10E+03	8,14E+01	1,00E+01	6,35E+00	5,69E-01		37,15	45,11	25,00
POD6 (MnP6)	1,62E+03	0	0	2,14E-02	0	0		0,08	0	0
POD7 (MnP7)	3,49E+04	8,70E+01	0	4,60E-01	5,00E-01	0		1,70	3,55	0
POD8 (LiP1)	1,35E+02	0	0	1,78E-03	0	0		0,01	0	0
POD9 (LiP2)	2,52E+03	0	0	3,33E-02	0	0		0,12	0	0
POD10 (LiP3)	1,32E+02	0	0	1,74E-03	0	0		0,01	0	0
POD11 (LiP4)	2,82E+02	0	0	3,72E-03	0	0		0,01	0	0
POD12 (LiP5)	7,76E+01	0	0	1,02E-03	0	0		0,00	0	0
POD13 (LiP6)	2,19E+02	0	0	2,88E-03	0	0		0,01	0	0
POD14 (LiP7)	1,93E+02	0	0	2,54E-03	0	0		0,01	0	0
POD15 (LiP8)	5,01E+02	0	0	6,60E-03	0	0		0,02	0	0
POD18 (LiP9)	7,18E+04	2,23E+02	0	2,92E-01	1,28E+00	0,00E+00		1,08	9,12	0
POD16 (VP1)	9,26E+01	0	0	1,22E-03	0	0		0,00	0	0
POD17 (VP2)	1,16E+06	1,03E+03	1,72E+02	1,53E+01	5,95E+00	1,20E+00		56,72	42,23	52,70
TUB (first set)	7,58E+04	1,74E+02	1,43E+02				Total	100,00	100,00	100,00

Comments:

- Data points are calculated as arithmetic means from three biological replicates.
- For all data points standard deviation of the mean is not higher than the half of least significant digit.
- Stability of the internal control gene (tubulin, TUB) expression under all experimental conditions was checked independently by RT-qPCR with several candidate genes. Data processing was done by GeneNorm software.
