# Supplementary material for: Lignin-degrading peroxidases in white-rot fungus Trametes hirsuta 072. Absolute expression quantification of full multigene family
Source: PLoS One. 2017 Mar 16;12(3):e0173813. doi: 10.1371/journal.pone.0173813 (PMC5354401; doi:10.1371/journal.pone.0173813)
Supplement: S3 Table — (PDF) [file pone.0173813.s006.pdf]

S3 Table. Identified secreted PODs from *Trametes hirsuta* 072.

Protein isozyme	Predicted isozyme	Max score	Max Expect	Calculated Mr, Da	Calculated pI	Unique peptides
POD2	MnP2	151	8.1e-008	38355	4.43	R.LDVFVGR.I<br>R.LQSDSELAR.D<br>K.TVPEPFDTVDS.I<br>R.GTLFPGTGGNQGEVESP.L
POD3	MnP3	69	2.1e-005	38390	4.70	K.MSSAFK.A<br>K.LAVIGQDVR.K<br>R.LQSDSELAR.D<br>R.GEFGGGGADGSISIF.E
POD4	MnP4	35	0.00032	38777	5.10	R.VSPDGLIPLPEDPVDK.I
POD5	MnP5	189	7.6e-017	38538	4.41	K.CCVWYDVLDDIQASDGLFQGGQCGEDAHQSLR.L<br>R.LTFHDAIGFSPALTSQGK.F<br>R.LQFLAGR.S<br>K.SPLVGEFR.M<br>K.FGGGGADGSIMAHSDVELTYGENFGMDDIVELQR.S<br>R.TACEWQSFVTDQASMVSK.F<br>K.FEAVMAK.L<br>R.SFALR.H<br>R.MTSDAELAR.D<br>K.LAVLGQDSSTLIDCSDVIPKPK.A<br>Q.VSPDNLVPSPADTVDAILDR.M<br>R.MSDAGFSAAEVVDLLASHSVAAQEHLDTTIVGSPLDSTPSVFDAQ.F
POD6	MnP6	123	8.5e-012	38672	4.82	R.LQSDFELAR.D<br>D.LTVPEPFDSVDK.I<br>R.DDIQANL.F<br>K.DATQPAPDLTVPEPFD.S<br>K.DATQPAPDLTVPEPFDSVD.K<br>K.DATQPAPDLTVPEPFDSVDK.I

POD7	MnP7	190	6.1e-017	38502	4.48	R.VACPDGVNTASNAACCALFPVLDDIQK.N<br>K.NLFDGGECGEEVHESLR.L<br>R.LTFHDAIGISPAISATGK.F<br>K.FGGGGADGSIAIFDDIETNFHANNGVDEIIGEQKPFIAR.H<br>R.LDVFVGRK.D<br>K.DATQPAPDKTVPEPFDTVDSILAR.F<br>K.TVPEPFDTVDSILAR.F<br>R.GTLFPGTGGNQGEVESPLHGELR.L<br>R.LQSDSELAR.D<br>R.TACEWQSFVNNQAK.L
POD17	VP2	152	3.8e-013	39382	4.52	R.ATCSGGQTTANDACCVWFDVLDDIQANLFHGGQCGEDAHESLR.L<br>R.VGDAGFAAVELVWMLISHTVAAQDK.V<br>R.LQSDFLIAR.A<br>R.TSCEWQK.M<br>K.MIIDR.A<br>R.ANMLQK.F<br>K.FEQTVLK.L<br>K.LSLLGFDK.S<br>K.STLTDCSDVIPR.A<br>R.ATGTVPDPFFPAGK.S
POD18	LiP9	42	5e-005	38315	4.41	R.LQSDFAIAR.D<br>F.HTPDQIFSR.L<br>R.LTFHDAIAISP.A<br>K.DATQPAPDGLVPEPFHTPDQIFSR.L<br>R.DDLQENL.F<br>K.DATQPAPDG.L<br>S.FVDNQEK.A<br>R.LTFHDAIA.I<br>R.DDLQENLFHGGL.C<br>K.DATQPAPDGLVPEPFHTPDQ.I

All proteins are glycosylated
