# Supplementary material for: Lignin-degrading peroxidases in white-rot fungus Trametes hirsuta 072. Absolute expression quantification of full multigene family
Source: PLoS One. 2017 Mar 16;12(3):e0173813. doi: 10.1371/journal.pone.0173813 (PMC5354401; doi:10.1371/journal.pone.0173813)
Supplement: S4 Table — (PDF) [file pone.0173813.s007.pdf]

S4 Table. ***Trametes hirsuta*** **072 secreted PODs during cultivation on different media.**

The number of isoforms found on electrophoregrams as counted with ImageMaster 2D Platinum v.7 program is mentioned within the cells.

Protein isozyme	Predicted isozyme	Cultural media/Days of cultivation								
		GP/3	GP/5	GP/8	BR/3	BR/5	BR/8	AL/3	AL/5	AL/8
POD1	MnP1									
POD2	MnP2	1		1	1		2	2		
POD3	MnP3						1			1
POD4	MnP4*									
POD5	MnP5*	11	4	4	9	14	13	11	11	10
POD6	MnP6		1	2			1		2	1
POD7	MnP7	1	1	1	1	1	1	1	1	1
POD8	LiP1									
POD9	LiP2									
POD10	LiP3									
POD11	LiP4									
POD12	LiP5									
POD13	LiP6									
POD14	LiP7									
POD15	LiP8									
POD18	LiP9*			2			1			2
POD16	VP1									
POD17	VP2*	4			6	6	2	4	6	2

*** The protein is additionally found on the AL medium on day 10**
